# The effects of verbal and spatial memory load on children's processing speed

**DOI:** 10.1111/nyas.13653

**Published:** 2018-04-30

**Authors:** Candice C. Morey, Lauren V. Hadley, Frances Buttelmann, Tanja Könen, Julie‐Anne Meaney, Bonnie Auyeung, Julia Karbach, Nicolas Chevalier

**Affiliations:** ^1^ School of Psychology Cardiff University Cardiff Wales United Kingdom; ^2^ Department of Psychology University of Edinburgh Edinburgh Scotland United Kingdom; ^3^ Department of Psychology University of Jena Jena Germany; ^4^ Center for Research on Individual Development and Adaptive Education of Children at Risk (IDeA) Frankfurt Germany; ^5^ Department of Psychology University of Koblenz‐Landau Landau Germany

**Keywords:** working memory, cognitive development, executive functions

## Abstract

Examining the impact of maintenance on processing speed allows us to test whether storage and processing resources are shared. Comparing these relationships in children of different ages allows further insight into whether one or multiple resources for these operations must be assumed and whether remembering is proactive throughout childhood. We tested 185 4‐ to 6‐ and 8‐ to 10‐year‐old children using adaptive complex span tasks, in which simple judgments were interleaved between to‐be‐remembered items. The adaptiveness of our tasks ensured that all participants frequently correctly recalled the items. If storage and processing require a single resource, and if participants serially reactivate the memoranda between processing episodes, processing response times should increase with serial position of the processing judgment within lists. We observed different within‐list dynamics for each age group. Older children's processing judgments slowed gradually when more than two memory items were maintained. By contrast, younger children showed no evidence of slower processing with increasing memory load. Our results support models of working memory that assume that some common resource is responsible for verbal and spatial storage and processing. They also support the notion that remembering becomes more proactive as children mature.

## Graphical abstract

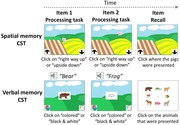

## The effects of verbal and spatial memory load on children's processing speed

Our ability to remember novel information even when we are interrupted is a seminal, benchmark finding that must be explained by models of working memory (WM). Evidence that storing memories and forming judgments may not interfere much with each other^1^ spurred new ideas about how attention and short‐ and long‐term memory may relate to one another. Still, dual‐task conflicts are frequently apparent, even in the midst of apparently successful multitasking.^2^ Modern models of WM disagree about how best to explain the concurrent storage and processing constantly occurring in complex cognition. Using data from a large sample of children, we compare the predictions of two prominent WM models developed to explain how storage and processing may be simultaneously carried out.

Multiple‐component WM models^3^ propose separate resources for storing verbal and visual–spatial information in addition to resources for controlling attention. Such models predict little or no interference between storage and processing operations,^4^ at least under certain conditions.^5^ In these models, some conflict is predicted between storage and processing when the sensory codes needed for mentally representing stimuli in both tasks overlap, particularly if the processing task also requires comparing a presented stimulus with a mental representation (e.g., rhyme judgments) or manipulating a mental representation (e.g., mental rotation^6^). However, when processing stimuli do not need to be mentally represented or if the sensory domains needed for representation in each task are distinct, no interference should occur between processing and storage.

Alternatively, many other models of WM assume that processes requiring attention rely on a common resource that may be used for maintenance of information.^7^, ^8^, ^9^ Though these models differ, they each predict that performing attention‐demanding processing operations will reduce the amount of information concurrently stored, and conversely that holding information in mind will impair accuracy or speed of processing. Empirical findings can be marshalled that concur with predictions of both kinds of WM model. Evidence of both independence^10^, ^11^ and conflict between storage and processing^2^, ^12^, ^13^, ^14^, ^15^ has been observed. Still more empirical work suggests that conflicts between storage and processing depend on other factors.^5^, ^16^, ^17^ Thus, models disagree in part because the evidence informing them seems contrary and incomplete.

Much of the evidence informing debate about how and when storage and processing operations interfere comes from complex span tasks (CSTs). CSTs measure how much one can remember when maintenance is interrupted by interleaved processing operations. As in a simple memory span task, participants are given a sequence of memoranda to be recalled in serial order. Between the presentations of each to‐be‐remembered item, participants must make some decision about an intervening stimulus, for instance, solving an arithmetic problem,^18^ judging whether a line would fit in between two markers,^19^ or judging the veracity of a sentence.^20^ CSTs may vary in many theoretically interesting respects, including the domains of the memoranda or processing task and the schedule of processing judgments to be made between memoranda. Spans from CSTs are consistently lower than those measured by simple memory span tasks with no processing interference, but this benchmark evidence may be explained in ways that include or exclude distinct WM resources for storage and processing.^14^ For instance, some multiple‐component models of WM^21^ and the time‐based resource sharing (TBRS) model^7^ both assume that memories decay over time and that elements of the WM system may be used to prevent loss of the representations, but nonetheless disagree about whether reactivating memoranda is a general process or one that depends on domain‐specific functions. If these maintenance operations are supported by general resources, we should observe not only effects of processing task on memory, but also effects of memory load on processing performance. If a common resource is used for carrying out demanding processing operations and for boosting memory strength, then increasing the amount of to‐be‐remembered material should slow processing judgments regardless of the domain of the memoranda.

Though investigations of CSTs usually focus on variations in memory performance while controlling processing task performance,^22^ some have taken the opposite approach and evaluated processing performance given retention of a variable memory load. In CSTs, processing responses are collected between presentations of memoranda, which conveniently creates a parametric memory load manipulation on processing judgments. While executing the processing task after the presentation of the first memory item, the participant can only store one item. After the second memory item, the participant stores two, and so on. As the list accumulates, the participant has increasingly more memoranda to retain and reactivate, which could produce an increasing burden on processing judgments. In a simple processing task unlikely to yield errors, this would manifest as slowing on the processing task with each memory item during a CST trial. There is some evidence for this pattern, but also some evidence against it. Maehara and Saito^23^ observed increased processing times (comparing the response times for the first and last processing judgment) in each of four possible combinations of verbal and spatial storage and verbal and spatial processing. However, Saito and Miyake^24^ observed an opposite, decreasing pattern on a reading span task, and Towse and colleagues^25^, ^26^, ^27^ observed decreasing or stable processing response times as a function of serial position in reading, operation, and counting span tasks. Null results could reflect insufficient power and cannot be easily interpreted, but, because the effect of storage on processing sometimes occurs in the unanticipated direction, these inconsistencies may reflect effects of moderating influences.

Two additional studies confirm that execution of the processing task depends on a variety of related factors that may obscure diagnostic patterns and limit interpretations of processing response times during CSTs. Engle *et al*.^18^ examined performance on reading and operation span tasks, including examining how processing speeds changed with memory load and individual differences in memory ability. Engle *et al*. found that effects of accumulating memory load on processing responses depended on which aspect of the processing task participants were performing. Their participants viewed each component of the processing tasks for as long as they chose. For instance, in operation span, participants looked serially at each element of the equation. When they were ready for the next element, they pressed a key to receive it. Participants could thus spend as long as they needed contemplating each element of the processing task and the to‐be‐remembered item. Engle *et al*. analyzed viewing times separately for the first element in the operation, the subsequent elements combined, and the memory item. They found that viewing times to the processing task elements increased with accumulating memory load, but only for participants with high WM span. When it came to viewing the memory item, voluntary viewing times generally increased for the first few items and then decreased, producing an inverted U‐shaped curve. Similarly, Friedman and Miyake^20^ observed significant increases in processing time on a reading span task as memory load accumulated for sequences up to four items. However, they also observed higher order effects in their data, and apparently did not observe clear, linearly increasing patterns when participants set the pace of the task. Taken together, these analyses of the effect of memory load on processing speed in CSTs suggest that memory load likely impairs processing, but perhaps not in a manner that can be captured by assuming a linear increase in response times with accumulating memory load.

Establishing whether (and under what conditions) accumulating memory loads impede processing during CSTs in children would benefit theories of WM development and of WM and executive functioning more broadly. Developmental trends can help distinguish among theories. If there are separate resources for storage and processing, these resources may show different developmental trajectories. By measuring CST performance in different age ranges, we can see whether observed differences among patterns of interference between storage and processing may be explained by supposing that components mature at different rates. If at least one component has not yet matured, then presumably conflicts between storage and processing would be greater for younger than older children. Some previous research suggests that the multiple components of the WM system are not set before age 7 or so,^28^ but recent developmental evidence suggests that the classic multicomponent WM model does not quite capture which functions rely on distinct resources.^29^, ^30^ Comparing the development of interference between storage and processing could conceivably strengthen multicomponent WM assumptions or suggest alternative explanations for WM maturation.

Processing task data from CSTs in children indeed present a somewhat different picture than in adults. Towse *et al*.^27^ examined effects of accumulating memory load on processing speed in samples of 6‐ to 11‐year‐old children who completed counting, operation, and reading span tasks. Despite similar research designs in each task, inferential results were thoroughly mixed, presenting every possible inferential outcome across the various studies. It is therefore plausible that processing response times increase or decrease with memory load, but also possible that they are uninfluenced by memory load. Age could potentially moderate this effect, though Towse *et al*. did not observe this. Barrouillet *et al*.^31^ observed that increasing the cognitive load of the processing task reduced observed memory spans. However, with younger children, any effects of cognitive load were attenuated. These findings suggest that there could be a point during development where memory and processing begin to conflict, which is difficult to explain by assuming the maturation of modules specialized for storage and processing; presumably, younger children lacking some specialized component that eases task performance should show more dual‐task interference, not less.

We present an analysis of the effects of accumulating memory load on processing task response times taken from two novel, adaptive CSTs. This large data set was collected as part of a preliminary study conducted to establish procedures for a study examining executive function training in school‐aged children. Here, we opportunistically extract the processing times from a 30‐min session of personally adaptive CST training, in which list length was determined per trial depending on the memory accuracy of the previous trial. The adaptiveness of our CST task ensures that every participant was performing our CSTs at challenging levels, where each would report the entire memory list correctly on ∼50% of trials. This affords a large sample of participants with ample number of trials to examine processing response times in which we can be sure that participants accurately retained the memoranda. We tested two groups of children aged between 48 and 72 or 96 and 119 months, and roughly half from each age group undertook a CST pairing visual processing with spatial memory, while half completed a task pairing visual processing with verbal memory. In both tasks, the processing judgment was to be performed on the memory stimulus itself, as in many previous studies.^20^, ^23^ This feature affords a naturalistic look at the effect of attempting to remember on processing; if children attempted to cumulatively reactivate previous memoranda with each new memory item, presentation of a separate processing stimulus might disrupt this activity.

This sample therefore offers us the chance to fully describe effects of memory on processing, so that we may detect and model fine‐grained effects of accumulating memory loads across the full spectrum of serial positions and possibly observe nonlinear trends, such as those reported previously in adults,^18^, ^20^ or confirm that memory loads do not affect processing speed in children.^27^ We also test whether accumulated memory load differentially affects processing in children under 7 and whether the domain of the memory load differentially affects processing in each age group. Both of these points potentially inform decisions about whether a unitary or multicomponent model of WM better explains the relationships between processing speed and concurrent memory load: The multicomponent view requires that memory load does not affect processing, or at least that a within‐domain memory load conflicts more with processing than a cross‐domain load, while the unitary models require that reactivating memory content slows processing. The multicomponent interpretation of WM development^32^ suggests that the verbal memory system matures later than age 7, thus predicting that children under 7 should show more interference between verbal memory and nonverbal processing than older children. Unitary WM frameworks, on the other hand, do not predict developmental differentiation toward different kinds of memoranda but are consistent with patterns showing that older children engage in active maintenance more than younger children.

## Method

### Participants

Study participants included 185 children in two age groups: 84 4‐ to 6‐year‐olds (M = 62 months, SD = 6.40; 33 females, 37 in the UK and 47 in Germany) and 101 8‐ to 10‐year‐olds (M = 107 months; SD = 6.6, 43 females, 52 in the UK and 49 in Germany). An additional 20 children were removed due to inability to operate the mouse independently, and an additional three were removed from the younger group because they never responded correctly on a trial with two or more memory items. Participants were recruited through university databases, schools, and advertisements on social media. Before participation, informed consent was obtained from the children's caregivers.

### Materials and procedure

Participants were tested individually by trained experimenters either in the laboratory or in a quiet room in their kindergarten or daycare in a single 90‐min session. Sessions began with a 22‐item language proficiency measure (the Peabody Picture Vocabulary Test (PPVT‐4)^33^) and a processing speed measure (color‐naming task^34^). Initial and final WM scores were then assessed in visuospatial and verbal memory tasks. These measures were collected for another project, which will be separately reported.

Participants performed the main CST experiment on Dell Precision 5510 laptops using the mouse to respond. The experiment was programmed in E‐Prime 2.0.^35^ The main CST training comprised four blocks of 12 trials, between which there were break periods that included watching videos and talking with the experimenter. Between trials, children saw two images and clicked on either to move on to the next trial. The CST trials comprised about 30 min of the session.

**Figure 1:**
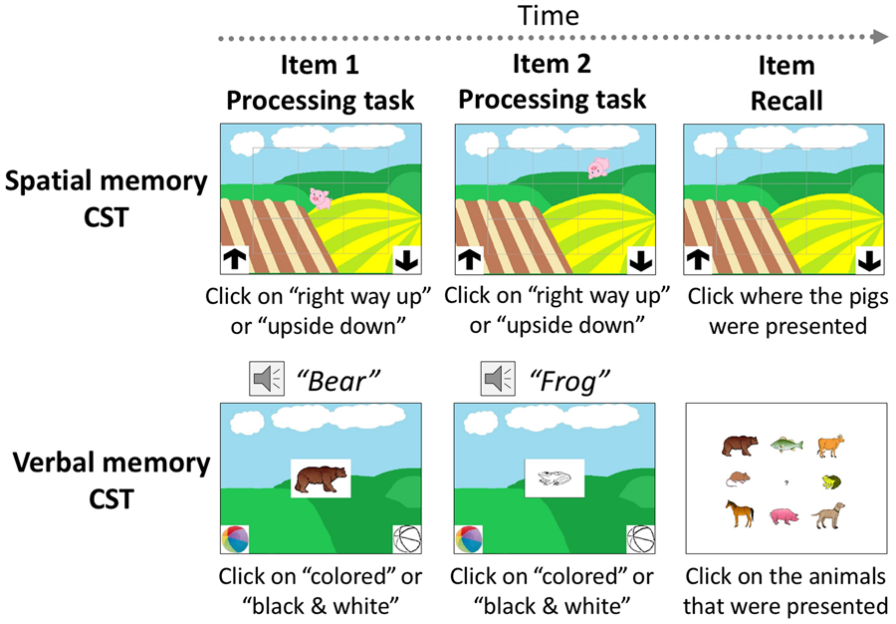
Example stimuli from the spatial and verbal memory versions of the complex span tasks. Images are not to scale.

The CST tasks are depicted in Figure 1. In the spatial memory condition, children observed a pig that was either the right way up or upside down placed within a to‐be‐remembered cell position on a 3 × 3 grid. The grid and pigs were overlaid on a field scene. Positions were selected randomly from the set of nine, and no positions were repeated within a trial. The child was to judge whether the pig was the right way up or upside down by clicking on either an up or down arrow image presented at the lower left and right corners of the screen. Progression to the next item occurred following the child's judgment about the pig's orientation. Immediately after the final processing judgment, the child was prompted to recall the grid positions presented on that trial by clicking them with the mouse in order. With each click, a gray dot was presented in the selected cell for 500 ms to indicate that the click had registered.

In the verbal memory condition, children remembered lists of animals. Drawings of animals were presented in the center of a field scene, with the names of the animals simultaneously presented aurally. Animals were chosen randomly from a set of eight (mouse, horse, pig, dog, frog, cow, fish, and bear) with single‐syllable names in both English and German, with none repeated within a trial. The animal images could be presented in color or grayscale. The child was to remember the animal name and judge whether the animal was in color by clicking either a colored or grayscale beach ball presented at the lower left and right corners of the screen. Progression to the next item occurred after the child's judgment about the animal's color. Immediately after the final judgment, an array including the set of eight animals was presented in the cells of a 3 × 3 grid, and the child was prompted to click the animals that were presented on that trial in serial order. The animals always appeared in the same cells within the grid, with the central cell left blank.

We used an adaptive procedure to ensure the task remained challenging. If the child got both the memory and processing responses correct, the trial length increased by one item. If they got the memory response correct but the processing task incorrect, they stayed at the same trial length, and if they got the memory response incorrect their trial length decreased by one item. Children were not given accuracy feedback and were not explicitly told how long each list would be, but they would have gradually learned that if the trial length changed; it differed by one item at most. Each new block began with the trial length of their prior trial.

### Design

We considered four factors of interest in addressing our theoretical questions: age group (4‐ to 6‐year‐olds and 8‐ to 10‐year‐olds), domain of the memory items (verbal or spatial), the trial length (ranging from 1 to 6 adaptively and thus differing per child), and the serial position (1 to trial length) within a trial. Trial length and serial position are both manipulations of mnemonic load on processing, but, because of the personalized adaptivity we employed, exact trial length is unbalanced across participants. We therefore focus on the effects of accumulating memory load on processing, which is reflected in the serial position factor. Analyzing processing responses as a function of serial position within a trial allows us to further test specific hypotheses about how storage and processing trade off. If participants engage in cumulative serial reactivation, then effects of memory load on processing should increase as the trial progresses, leading to ever‐slower processing response times. We estimated Bayesian repeated‐measures analysis of variance (ANOVA) models^36^ using the BayesFactor^37^ R package (version 0.9.12‐2, using the default settings), comparing Bayes factors for models excluding versus including these four terms. This approach allowed us to quantify evidence both for and against particular effects and interactions in our data.

## Results

### Preliminary data processing

We took care to ensure that the data we included in our analyses were likely to reflect legitimate processing decision activity. As described above, we excluded data from participants who were unable to personally operate the mouse. We then excluded responses in which we detected that the child erroneously clicked an invalid location before clicking the intended spot. Our programs did not mark when a mis‐click occurred, but we could detect most instances of invalid clicks by comparing recorded response times against deduced response times calculated from known onset times of trial events. We lost ∼7% of trials due to detection of an invalid click. As recommended by Vergauwe *et al*.,^15^ we also restricted analyses of the processing task to trials in which participants reported all the memoranda in correct order. However, because of our adaptive procedure, we acquired similar, ample amounts of data from each participant.

Our Bayes factor modeling was performed on per‐participant averages of processing accuracies and per‐trial log‐transformed processing response times. Accuracy averages were used for the convenience of obtaining continuous values. For response times, analyzing trial‐level responses was important to avoid distorting effects of serial position, which should not be conflated across different trial lengths. We report the Bayes factors for the models that emerge from each analysis with the strongest support and then evaluate the presence or absence of terms of interest by comparing the model including or excluding the term of interest with the best model. We invert Bayes factors less than 1 so that we consistently express support for or against including the term in integers.

### Memory spans

Table 1 provides a summary of memory span scores showing the average maximum trial length reached with at least one correct list recalled, organized by age group and memory domain in order to describe memory performance on our tasks. A Bayes factor *t*‐test comparing the maximum trials lengths reached by participants in the verbal and spatial memory groups did not reveal any evidence of a difference (inverted BF = 5.56).

**Table 1:** Summary of memory spans by age group and memory domain

	Mean (SD)	Min.	Max.	*n*
4‐ to 6‐year‐olds				
Verbal memoranda	2.88 (0.75)	2	4	41
Spatial memoranda	2.77 (0.65)	2	4	43
8‐ to 10‐year‐olds				
Verbal memoranda	4.88 (0.72)	3	6	50
Spatial memoranda	5.20 (0.78)	3	6	51

### Processing task accuracy

**Figure 2:**
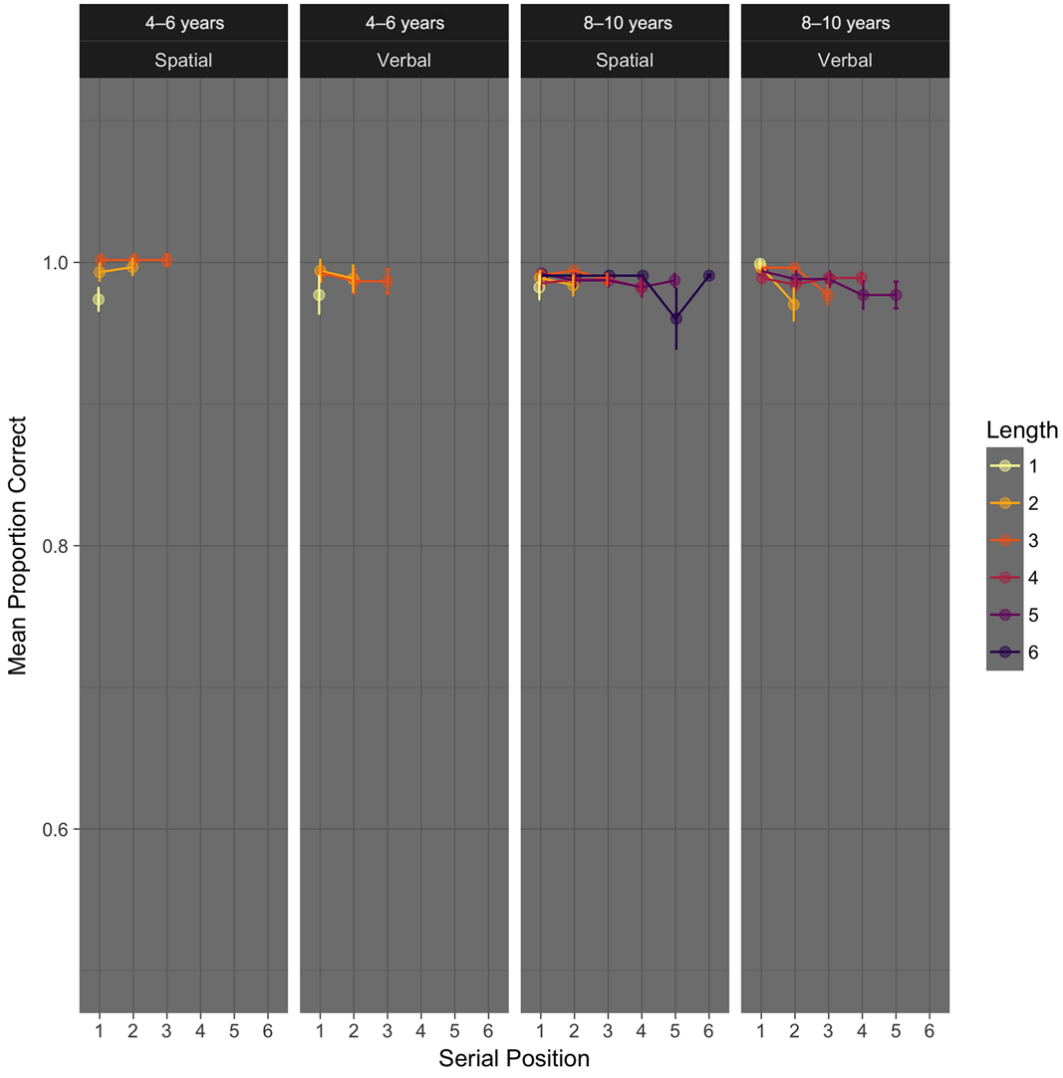
Normalized processing task accuracy for trials with 100% correct recall of the memoranda. Error bars are within‐participant standard errors of the mean, calculated with the Cousineau–Morey^54^ method. *N* = 185 overall, with 43, 41, 51, and 50 per panel (left to right).

In both age groups and regardless of memoranda domain, participants had no trouble making accurate decisions on the processing tasks. Figure 2 shows processing task accuracy as a function of age group and memory domain (separated by panel), serial position of the processing judgment in the trial (*x*‐axis), and overall trial length (separated by color). We ran a four‐way Bayes factor ANOVA on processing task proportions correctly (subjected to arcsine square root transformation), including each factor in our design. This analysis allowed us to rule out effects of trial length or serial position, as well as any interaction involving these factors, on processing task accuracy. Excluding these terms from the complete model was preferred by factors of at least 8 (and as much as 889). We looked at potential effects of age group and memory domain more closely in a two‐way ANOVA, because the omnibus analysis suggested that these factors might have an impact. The model with the highest BF included a main effect of age group only (BF = 41.94 ± 1.30%). The models that also include memory domain and the interaction between age group and memory domain could not be definitively ruled out (the BFs for excluding these terms were 1.28–1.58). If anything, 4‐ to 6‐year‐old children performed less well overall with verbal (M = 0.97, SD = 0.12) than with spatial memoranda (M = 0.99, SD = 0.06), while 8‐ to 10‐year‐old children performed equally well in both memory conditions (M_Verbal_ = 0.99, SD = 0.03; M_Spatial_ = 0.99, SD = 0.06). Clearly, performance was at ceiling in each case. These few errors were excluded from the analysis of response times.

### Processing task response times

We trimmed response times for further analysis using the R package *trimr* 38 (version 1.0.1), which trims outlier response times according to the method of van Selst and Jolicoeur.^39^ In addition to the exclusions described above, we further excluded incorrect processing task responses and responses slower than 9000 ms before implementing a trimming procedure in which responses faster than 200 ms and 2.5 SDs slower than the mean per participant were eliminated. Finally, we excluded responses of trial lengths greater than 5 in 8‐ to 10‐year‐olds and greater than 3 in 4‐ to 6‐year‐olds because there were very few trials at these levels. Using this procedure, we excluded about ∼5% of otherwise valid data.

**Figure 3:**
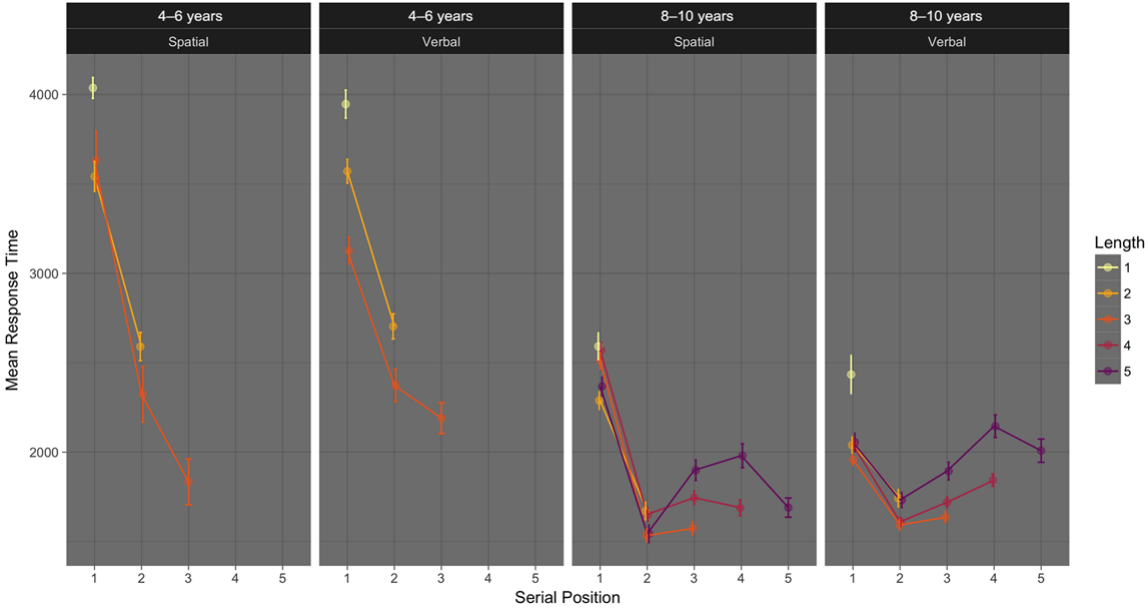
Processing task response times for trials with 100% correct recall of the memoranda and accurate processing responses. Error bars are within‐participant standard errors of the mean, calculated with the Cousineau–Morey^54^ method. *N* = 185 overall, 43, 41, 51, and 50 per panel (left to right). Each mean is based on at least 55 observations.

Figure 3 depicts these data. Most obviously, the slow response times at serial position 1 are contrary to both models’ expectations but not completely unprecedented^40^ (we return to this in the discussion below). To begin, we ran a four‐way Bayesian ANOVA on log‐transformed response times. The best model (BF = 6.54 × 10^455^ ± 2.35%) included each main effect, a three‐way interaction between age group, memory domain, and serial position, and two‐way interactions between age group and memory domain and each factor with serial position. This model was favored decisively over the next best model (which included more terms) by a factor of 135. Including the three‐way interaction was even more decisively favored (BF > 23,000), which justifies analyzing data from each age group separately in two more interpretable three‐way ANOVAs. We turned immediately to those.

#### Processing task response times, 4‐ to 6‐year‐olds

The best model included only main effects of trial length and serial position (BF = 5.57 × 10^124^ ± 1.70%). Inclusion of both terms was decisively favored (BFs > 121), and exclusion of their interaction was favored by a factor of 5.84. Both trial length and serial position represent memory load, which suggests that memory load affected processing speed in 4‐ to 6‐year‐olds. However, any effects of trial length or serial position in this group go against expectations. Response times obviously decreased with each serial position (Fig. 3). Also, response times became faster with increasing trial length (M_1_ = 4004, SD = 1602; M_2_ = 3093, SD = 1588; M_3_ = 2563, SD = 1398). Because of our adaptive procedure, trial length differences may largely reflect speed differences among children; individuals would not all have contributed equally to means for each trial length. However, both findings are inconsistent with the idea that memory load impairs processing task performance in children younger than 6.

Exclusion of an effect of memory domain was favored by a factor of 6.46, and exclusion of the interaction between memory domain and serial position was favored by a factor of 3.51.

#### Processing task response times, 8‐ to 10‐year‐olds

The winning model included main effects of trial length, memory domain, and serial position, plus interactions between trial length and serial position and memory domain and serial position (BF = 1.47 ×10^299^ ± 3.18%). Inclusion of each term in this model was decisively favored by factors of more than 1 million.

Because trial length varied among participants and is thus difficult to interpret, we focus on the interaction between memory domain and serial position. To diagnose the meaning of this interaction, we carried out separate series of ANOVAs for verbal and spatial memoranda conditions. Within each, we compared alternative codings of serial position to allow for four possibilities: (1) that only the first serial position differed from the others; (2) that the first and fifth serial position differed from the second, third, and fourth; (3) that serial positions 1 and 2 were unique, and other positions were the same; and (4) that all serial positions differed. We always included trial length in the modeling to ensure that serial positions from different trial lengths remained distinct. With verbal memoranda, the best model required the most complex serial position coding in which each serial position differed (BF = 6.49 ×10^74^ ± 1.66%). This model was decisively favored over the next‐best simpler serial position model by a factor of more than 18,000. This means that, with verbal memoranda, from serial position 2 onward, processing times increased incrementally, as one would expect if participants cumulatively refreshed the verbal memoranda, and if these maintenance operations impinge on the same resource needed for processing (Fig. 3). For spatial memoranda, it was less clear whether each serial position differed. Considering the same possibilities, the best model likewise included the serial position coding in which each position differed (BF = 6.93 ×10^194^ ± 0.93%), but this model was only slightly favored (by a factor of 3.39) over a model including the simpler serial position coding in which positions 3, 4, and 5 do not differ from each other. From Figure 3, it is clear that processing task response times consistently increase with verbal memoranda from position 2 onward, whereas with spatial memoranda they may not. Though Figure 3 appears to show that processing times increase with spatial memoranda at the longest list length, we did not observe the interaction among memory domain, trial length, and serial position that would allow us to interpret this clearly.

#### Analysis of trial‐length 3 including both age groups

Our adaptive procedure ensured that participants of all ages were performing the CSTs at a level of performance well off both floor and ceiling. Achieving this naturally means that younger children receive shorter lists than older children. To check that the apparent differences between 4‐ to 6‐ and 8‐ to 10‐year‐old children are not only due to the older children being able to recall more and reach longer trial lengths, we repeated our analysis on the data from both groups from trial length 3, the longest common length across groups for which we obtained sufficient data. The best model included effects of age group, memory domain, and serial position, plus interactions between age group and serial position and memory domain and serial position. The interaction between age group and serial position was favored by a factor of more than 400, which confirms the idea that interlist dynamics differ by age. However, this should be interpreted cautiously: response times were much slower overall in the 4‐ to 6‐year‐olds, and three items was the minimum span achieved by the older children and thus was not challenging for most 8‐ to 10‐year‐olds.

## Discussion

We analyzed response times for the processing components of CSTs recorded while participants correctly maintained verbal and spatial memoranda. In 4‐ to 6‐year old children, memory load did not slow processing task response times. In 8‐ to 10‐year old children, we observed a complex pattern of results: while responses on the first processing judgment in a trial were consistently the slowest, processing judgments slowed from the second item onward when the memoranda were verbal. With spatial memoranda, processing judgments also tended to slow from the second onward, but the pattern was somewhat less compelling. Our data confirm that effects of memory load on processing emerge between 6 and 8 years of age, which is consistent with other theoretical accounts of WM development.^31^

Our findings contradict the notion that domain‐specific short‐term storage systems are developing in this period. According to the classic multicomponent WM account, storage and processing should be distinct, and memory load should not affect processing. Even if we relax this assumption by allowing that processing might involve briefly representing to‐be‐processed stimuli in a short‐term store and thereby provoking interference, one might expect to observe within‐domain interference between spatial storage and visual processing, where spatial memory load increases visual processing task response times, but verbal memory load does not. In younger children, we observed decisive evidence against any difference between verbal and spatial mnemonic load on visual processing, not the dissociation that would be expected if visual–spatial information were maintained in a dedicated short‐term store. If one sort of store was undergoing development and not yet functional, we should have seen more evidence of interference in younger children with underdeveloped WM components, not less evidence of interference, as we clearly did. In older children, we observed evidence that memory loads impaired processing and interactions showing that verbal and spatial mnemonic loads differentially affected processing. However, the evidence that increasing verbal load resulted in slower processing was stronger than the evidence for the analogous effect with spatial load. These effects are incompatible with the domain‐specific short‐term store assumptions, because, if anything, within‐domain interference should have been greater than between‐domain interference. Our results are more compatible with accounts that clearly acknowledge that general processes are used in the service of verbal and spatial WM storage.

One interpretation of the development of multicomponent WM across childhood is that the verbal rehearsal component is only fluently applied after age 7.^28^, ^41^ Some evidence suggests that, before the emergence of verbal rehearsal, children rely strongly on representing information in visual code, avoiding converting nameable picture stimuli to verbal labels, as older children seem to do.^42^, ^43^ In our results, older children's slowed processing responses with increasing numbers of verbal memoranda are consistent with the idea that only children over age 7 attempt to reactivate verbal memoranda during processing. However, we see an alternative explanation that fits well with our findings and with previously published evidence. Rather than frame younger children's behavior in terms of a preference for using visual WM, one could explain the absence of verbalization of memoranda in children under 7 years of age using the dual mechanisms of cognitive control framework,^44^ which suggests that individuals have recourse to two cognitive styles—one in which they proactively prepare to respond before receiving a prompt and one in which they formulate their response reactively when the response is requested. Perhaps children begin to implement proactive control around 7 years of age. In a memory task, proactivity presents as any behavior that reflects attempts to keep the memoranda in mind before prompted to recall,^45^, ^46^ including applying verbal rehearsal. Under this model, children under age 7 do not verbally recode or rehearse, but this need not imply that they are not capable of doing so or that they lack the cognitive resources to do so. Barrouillet *et al*.^31^ invoke a similar explanation to account for the absence of cognitive load effects in children under age 7. The lack of slowing we observed with accumulating memory load in 4‐ to 6‐year‐old children is expected under the dual‐mechanisms framework, because children of this age are not assumed to be proactively trying to think of the memoranda during the processing task at all, no matter how long the list is. If participants do not attempt to reactivate memoranda, they will not experience an effect of mnemonic load. Our data similarly suggest that 8‐ to 10‐year‐old children prepare more with longer lists, which is hinted in the slower responses for a given serial position when it is part of a longer trial length (a pattern also observed in adults in Brown–Peterson–style storage‐plus‐processing tasks^15^, ^40^). Invoking the dual‐mechanisms account does not necessarily dispel the multicomponent or TBRS assumptions about WM structure but presents a viable explanation that may limit how task performance data can be used to infer structure. We think that considering the dual‐mechanisms framework provides a way to interpret developmental change in memory without presuming that some specific component of WM is absent or dysfunctional before age 7.

Aspects of our findings are also consistent with the predictions of the TBRS model. TBRS^7^ assumes that storage and processing require the same attentional resource, predicting that storage operations will slow concurrent processing and that processing will likewise prevent maintenance operations from occurring. As the to‐be‐remembered items accumulate, processing times should become slower, because participants are devoting the central resource to maintaining more memoranda. Younger children are assumed to not attempt covert maintenance during processing tasks,^31^ so 4‐ to 6‐year‐old children may not respond more slowly as mnemonic load increases. In the verbal condition from serial position 2, we observed this predicted increase in 8‐ to 10‐year‐old children. With spatial memoranda, this linear increase with serial position was less obvious but apparently present. However, to make the TBRS predictions fit our data, we must ignore the long processing times for item 1 and the apparent limits on the assumption of linear increases in processing time with memory load (i.e., processing times do not seem to continue rising indefinitely). The robust patterns we observed are consistent in some important ways with TBRS, but also deviate from the TBRS predictions and may be used to modify the account.

The slowest processing responses consistently occurred for the first processing item, a pattern not predicted by either WM model we consider in depth here or any other model of which we are aware. It may be tempting to disregard this observation, but we are not the only ones to report it,^40^ and it may have been present in other data sets but obscured in data sets where difference scores were analyzed. First responses may differentially reflect a switch from the preblock task instructions. In Brown–Peterson–style interference paradigms, the first processing response is the slowest, and this is attributed to switching from encoding memoranda to processing novel stimuli.^15^, ^47^ However, when applied to CSTs, this explanation is unsatisfying, because participants are constantly switching between encoding and processing. In our CSTs, this slowing could reflect trials at the beginning of blocks in which participants switch from listening to instructions and chatting with the experimenter to performing the task. However, we liberally removed long response times and additionally trimmed response times based on per‐participant means and standard deviations. If some trials got off to a slow start as the children transitioned from the interblock training instructions to the CST, we would expect these responses to be among those removed in our trimming procedure. Children were encouraged to reflect on their performance at the end of each trial, and first‐item slowing could reflect a transition from this reflection to recommencing the task. However, though most previous research using comparable methods did not report such a large speeding between processing judgments 1 and 2, many of these studies did not report their data in sufficient detail to know whether a similar pattern occurred. Frequently, effects of memory load on processing were expressed as differences in processing speed between the first and last item. This approach tells us nothing about whether the intermediate responses were quicker than the first response. The reports that provided fine within‐list detail^18^, ^20^, ^40^ acknowledged more complex patterns in their adult samples than a mere linear increase with processing task position. It may be that encoding the first memory item in a set is special. Encoding memory item 1 out of *n* might entail not merely representing a single item, but also establishing the representational object that the remainder of the list will eventually populate. Though we can only speculate as to why we may have observed these slow processing responses to the first memory item, it may be that the temporal context of the first item in a list is unique from the others. Models of memory frequently attempt to account for this (see summaries of models described by Hurlstone *et al*.^48^). We therefore think that we cannot completely dismiss the long response times we observed to the first processing judgments, but, given the novelty of this observation in children, we can only speculate about their source. Despite the long response times to the first processing judgments, which presumably occur with loads of only one memory item, we think that TBRS is essentially correct in supposing that the same resource needed for the processing task may also be used to boost maintenance. This account is compatible with the idea that individuals differ in their use of cognitive resources in the service of any task.^45^, ^49^, ^50^ However, we also think that it may be time to generate more refined predictions about how boosting memory via reactivation is likely to play out across serial lists. The assumption that processing response time increases linearly with memory load is too simple for our findings (and for previous findings^20^, ^40^). We have already noted that encoding the first memory item may well differ from encoding the remaining items; we think this merits further consideration. Considering memory reactivation as a function of the end‐of‐list context also seems important. One would not necessarily expect participants to bother with strategic covert maintenance immediately before recall; it might be reasonable to assume that processing response times at the end of the list would be less affected by memory load than those in the middle of the list. The complex patterns that we observed, as well as those documented by others,^18^, ^20^ might help generate boundary conditions on assumptions about when covert maintenance might be prioritized in CSTs. While the simplicity of assuming a linear increase in processing response times with memory load is attractive, it is entirely plausible that temporal context and limits to how many memoranda may be covertly reactivated during any processing episode justifiably limit the explanatory power of the assumption of a linear effect of memory load on processing speed.

Finally, we acknowledge that the accounts we compared were not the only viable accounts of CST performance. Many other models of WM may be called upon to explain CST performance. The multicomponent and TBRS models make differing predictions about phenomena we could address given the variables we manipulated for a different purpose. With these tasks, we were unable to fully differentiate maintenance processes acting to strengthen memoranda from processes that may act to inhibit or remove distractors, which leaves us unable to comment much on that contemporary discussion.^51^ Either sort of process could lead to serial position effects on processing response times. However, we surmise that an account of CST performance that replaces reactivation of memoranda with removal of distractors would likewise struggle to fully account for the patterns we observed. The slow responses to the first processing judgment are equally problematic regardless of which maintenance‐related activity we assume occurs between the first and second item, when there is only one item to remember (and correspondingly one feature to remove or inhibit). Additionally, because our processed stimuli were aspects of the memoranda themselves, it is not clear whether removal of the processed feature would serve to reduce interference in the same manner as removing a separate item that may erroneously become bound to the same temporal position as the memory item. Because maintaining multifeature objects costs more than maintaining single‐feature objects,^52^, ^53^ maintenance of processed features might have reduced memory spans overall if the irrelevant feature were not removed, but we have no means to check this in these data. While our results clearly suggest that both verbal and spatial memory loads slow visual processing from the age of 8 or so, we cannot claim this occurs because of the development of a particular kind of maintenance operation.

Despite these caveats, these data uniquely portray how accumulating verbal and spatial memory lists affect an interleaved processing task throughout to‐be‐remembered lists in 4‐ to 10‐year‐old children. Previous investigations of CSTs in this age range^27^ did not present the full patterns of processing task data we present, and we know of no other CST investigation with children that makes use of adaptive list length selection to ensure an appropriate balance between participants’ effort and accuracy. Our data will therefore be very useful in advancing discussion of how WM is limited and how it develops across childhood. Any complete account must acknowledge and explain conflicts between maintenance and processing operations and the emergence of these conflicts by age 8.

## Competing interests

The authors declare no competing interests.
